# Online Discussion on #KidneyStones: A Longitudinal Assessment of Activity, Users and Content

**DOI:** 10.1371/journal.pone.0160863

**Published:** 2016-08-18

**Authors:** Johannes Salem, Hendrik Borgmann, Matthew Bultitude, Hans-Martin Fritsche, Axel Haferkamp, Axel Heidenreich, Arkadiusz Miernik, Andreas Neisius, Thomas Knoll, Christian Thomas, Igor Tsaur

**Affiliations:** 1 Department of Urology, University Hospital, Cologne, Germany; 2 Department of Urology, University of Medicine, Mainz, Germany; 3 Stone Unit, Guy's and St. Thomas' NHS Foundation Trust, London, United Kingdom; 4 Department of Urology, Caritas St Josef Hospital, University of Regensburg, Regensburg, Germany; 5 Department of Urology, University Medical Center, Freiburg, Germany; 6 Department of Urology, Klinikum Sindelfingen-Böblingen, Sindelfingen, Germany; Emory University Department of Medicine, UNITED STATES

## Abstract

**Introduction:**

Twitter is a popular microblogging platform for the rapid dissemination of information and reciprocal exchange in the urological field. We aimed to assess the activity, users and content of the online discussion, #KidneyStones, on Twitter.

**Methods:**

We investigated the Symplur Signals analytics tool for Twitter data distributed via the #KidneyStones hashtag over a one year period. Activity analysis reflected overall activity and tweet enhancements. We assessed users’ geolocations and performed an influencer analysis. Content analysis included the most frequently used words, tweet sentiment and shares for top tweets.

**Results:**

3,426 users generated over 10,333 tweets, which were frequently accompanied by links (49%), mentions (30%) and photos (13%). Users came from 106 countries across the globe and were most frequently from North America (63%) and Europe (16%). Individual and organisational healthcare professionals made up 56% of the influencers of the Twitter discussion on #KidneyStones. Besides the words ‘kidney’ (used 4,045 times) and ‘stones’ (3,335), ‘pain’ (1,233), ‘urine’ (1,158), and ‘risk’ (1,023) were the most frequently used words. 56% of tweets had a positive sentiment. The median (range) number of shares was 85 (62–587) for the top 10 links, 45.5 (17–94) for the top 10 photos, and 44 (22–95) for the top 10 retweets.

**Conclusion:**

The rapidly growing Twitter discussion on #KidneyStones engaged multiple stakeholders in the healthcare sector on a global scale and reached both professionals and laypeople. When used effectively and responsibly, the Twitter platform could improve prevention and medical care of kidney stone patients.

## Introduction

The microblogging social media platform, Twitter, enjoys increasing popularity in the healthcare sector. Currently, over 70% of urologists in Australia and New Zealand have a social media presence, with Twitter being the second most commonly used form after LinkedIn, which serves a completely different purpose [1]. Urologists using Twitter during the European Association of Urology (EAU) and the American Urological Association (AUA) congresses regarded it as beneficial for professional networking, disseminating information, research, advocacy, and career development [2]. Impressively, 1,860 users generated 15,419 tweets in total at the EAU14 and AUA14 congresses [3]. Notably, the Twitter-based International Urology Journal Club, #urojc, has established a high-level academic discussion of urologic manuscripts [4]. In attempt to standardise the online discussion about urological care, a particular structuring of the key urology-related hashtags has recently been proposed [5]. Interestingly, the high technology field of endourology was the first urologic subspeciality assessed for Twitter activity during the 2013 World Congress of Endourology [6]. Most recently, a Twitter discussion during the Third Meeting of the European Association of Urology Section of Urolithiasis 2015 comprised 94 users contributing 446 tweets [7].

In slightly more than a decade, the prevalence of urolithiasis in the United States has increased from 5.2% to 8.8% [8], having a substantial socioeconomic impact [9] and incrementally affecting younger patients [10]. Since evidence has been provided for both highly prevalent urologic conditions and also diseases with young patient age at onset evoking more Twitter activity than their counterparts [11], a vibrant Twitter discussion on stone disease is likely. Interestingly, in conditions with broad-based Twitter communities, such as breast cancer, patients have reported an increase in disease knowledge from participating in Twitter discussions [12].

In the current investigation, we aimed to assess the potential of Twitter to constitute a potential platform for the dissemination of contemporary evidence on prevention, diagnosis and treatment of stone disease. Hypothesising that a Twitter discussion on #KidneyStones might appreciably involve both healthcare givers and laypeople, as well as encourage considerable global public awareness, we investigated the characteristics of its activity, users and content.

## Materials and Methods

The study was approved by the Ethics Commission of the Faculty of Medicine of the University of Cologne. We performed an extensive analysis of activity, users and content of the online discussion on #KidneyStones on Twitter, using the Symplur Signals database. Symplur (www.symplur.com) is a Twitter analysis website that maintains a database of healthcare-related Twitter conversations. Symplur Signals (www.symplur.com/signals) is a fee-based research analytics tool that promotes the understanding of healthcare as seen by patients, doctors and other stakeholders with access to healthcare social media data points.

In October 2015, we searched the Symplur Signals database for analytic insights into the online discussion on #KidneyStones for the time period 1st October 2014 to 1st October 2015. The activity analysis comprised the assessment of overall tweet activity, tweet metrics, engagement metrics and tweet language metrics. Overall tweet activity was recorded as the number of tweets and users, and these were related to time periods. We performed a detailed analysis of the tweet transcript (exact list of all tweets) to assess the issues inducing peak activities. The tweet metrics analysis was performed by retrieving statistics about ratio and frequency of retweets, tweets with links, tweets with photos, tweet replies and tweets where one or more Twitter users were mentioned. Engagement metrics were retrieved by obtaining the number of users who tweeted over a set period of time, grouped by the number of tweets sent. A tweet language analysis illustrated the language used by active participants over a set period of time. Language type was identified by a natural language processing algorithm directly provided by the Twitter application programming interface.

User analysis included the cumulative user report, users’ geolocations, and an influencer analysis of the top influencers in the #KidneyStones discussion. Cumulative and new users were recorded for monthly intervals. Cumulative users did not represent recurring users, but were counted as a new user the first time they used the #KidneyStones hashtag and as a previous user in subsequent reporting periods, regardless of activity. The geolocation of users was recorded when users sent tweets tagged with certain geolocation data. We analysed the top 100 contributors to the #KidneyStones discussion, as measured by number of tweets in the influencer analysis. For this purpose, we performed a Twitter profile analysis and assigned these top influencers to these healthcare categories, in line with the Symplur Signals healthcare category definitions: physician; patient; healthcare professional; caregiver/advocate; researcher/academic; individual other healthcare; individual other non-healthcare; organisation provider; organisation research/academic; organisation government; organisation advocate/support; organisation pharma; organisation other healthcare; organisation other non-healthcare, and spam [13].

We used the Symplur Signals tools for content analysis. The 100 most frequently used words in tweets on #KidneyStones were analysed and counted. Since multiple hashtags are often used within a single tweet, we used the hashtag network graph to analyse hashtags accompanying the #KidneyStones discussion and their relationships. The sentiment report analysed tweets for positive and negative sentiment by a natural language processing algorithm. The algorithm is based on two custom dictionaries, one for positive words and one for negative words. Each word in the dictionaries has a weighting from one to five, with five being the highest. Finally, we investigated the most frequently shared links, photos and the most frequently retweeted tweets. We performed statistical calculations using the Statistical Package for the Social Sciences 22.0 software (SPSS Inc., Chicago, IL, USA). Values are described as median and range.

## Results

**Fig 1:**
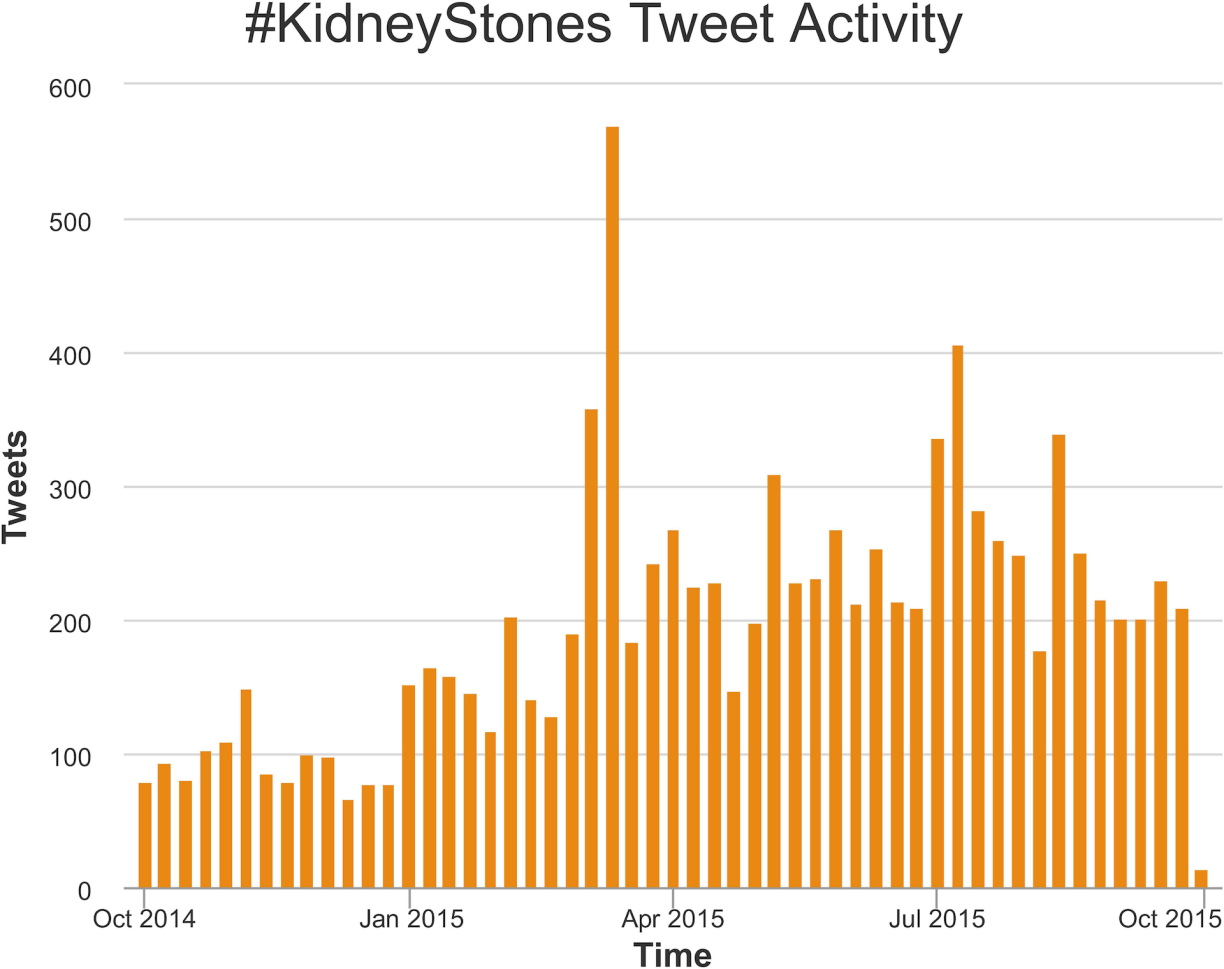
Tweet activity for the #KidneyStones online discussion on Twitter during the one year period. The weekly numbers of tweets are represented as columns.

Table 1 shows overall activity for the #KidneyStones online discussion on Twitter for the time period of one year. 3,426 users produced 10,806 tweets. Fig 1 portrays the weekly number of tweets on #KidneyStones, which slightly increased over the investigated time period. The peak tweet activity occurred during a strategic massive tweet activity of key influencers, evoking a large amount of retweets (influencers: @virtualclinicng, 9th March 2015 and 11th March 2015, 41 tweets on kidney stones prevention leading to a peak of over 500 tweets in a week; @mayoclinic, 11th July 2015, 41 tweets on kidney stones prevention during a radio show leading to a peak of over 400 tweets in a week).

Out of 10,333 total tweets, 5,013 (49%) were accompanied by links, 3,101 (30%) by mentions and 1,337 (13%) by photos. 2,441 (24%) were retweets and 229 (2%) were tweet replies. 2,798 users (82%) generated one tweet, 331 (10%) two, 244 (7%) three to nine, and 53 (2%) 10 or more tweets. 9,942 (96%) of tweets were in the English language.

**Table 1:** Overview of tweet activity for the #KidneyStones online discussion on Twitter over a time period of one year.

Metric	Total	Per Month	Per Week	Per Day	Per Hour
Tweets	10,333	849	198	28.3	1.18
Tweets per user	3.02	0.248	0.0578	0.00826	0.000344
Users who tweeted	3,426	282	65.7	9.39	0.391

**Fig 2:**
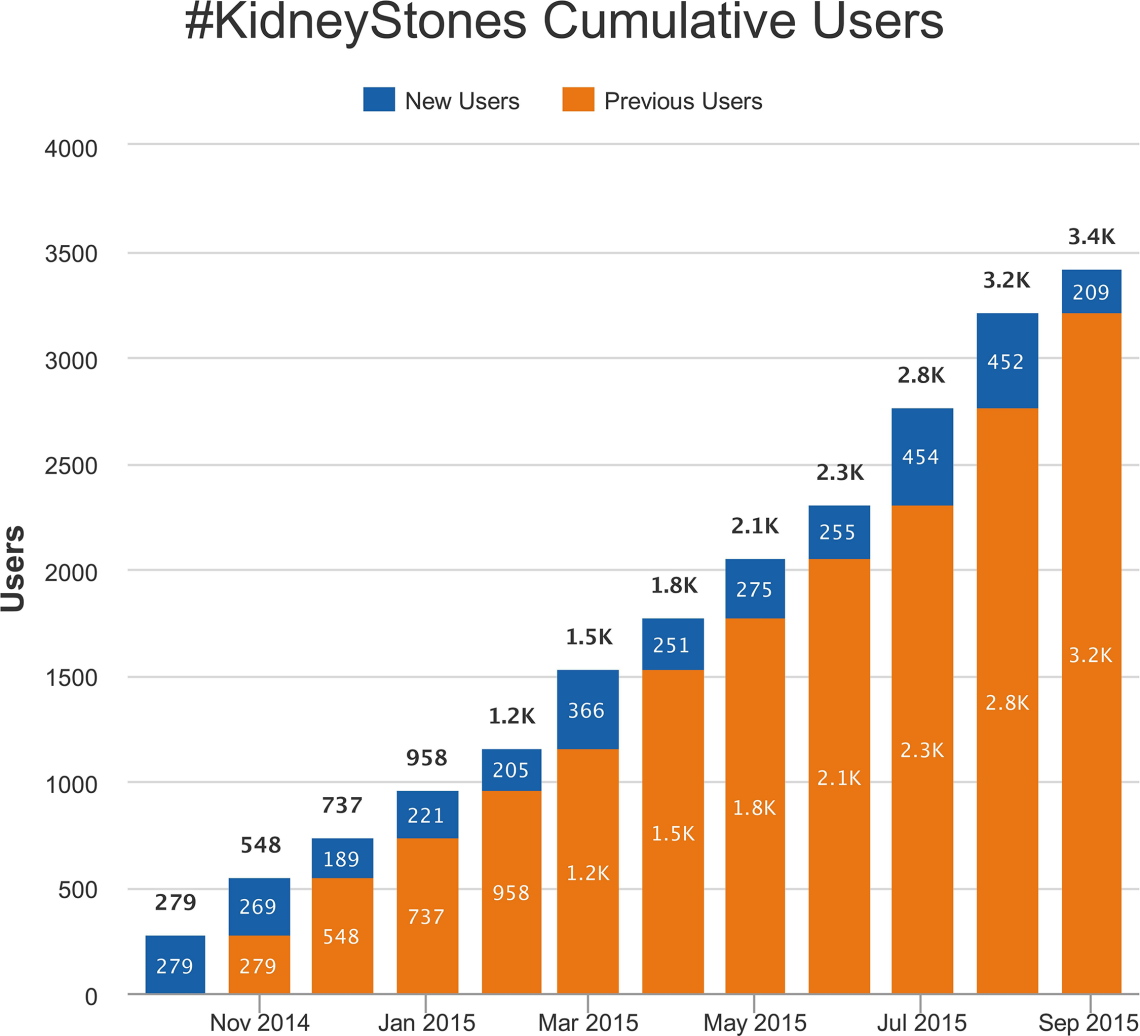
Growth in number of users contributing to the #KidneyStones online discussion on Twitter.

**Fig 3:**
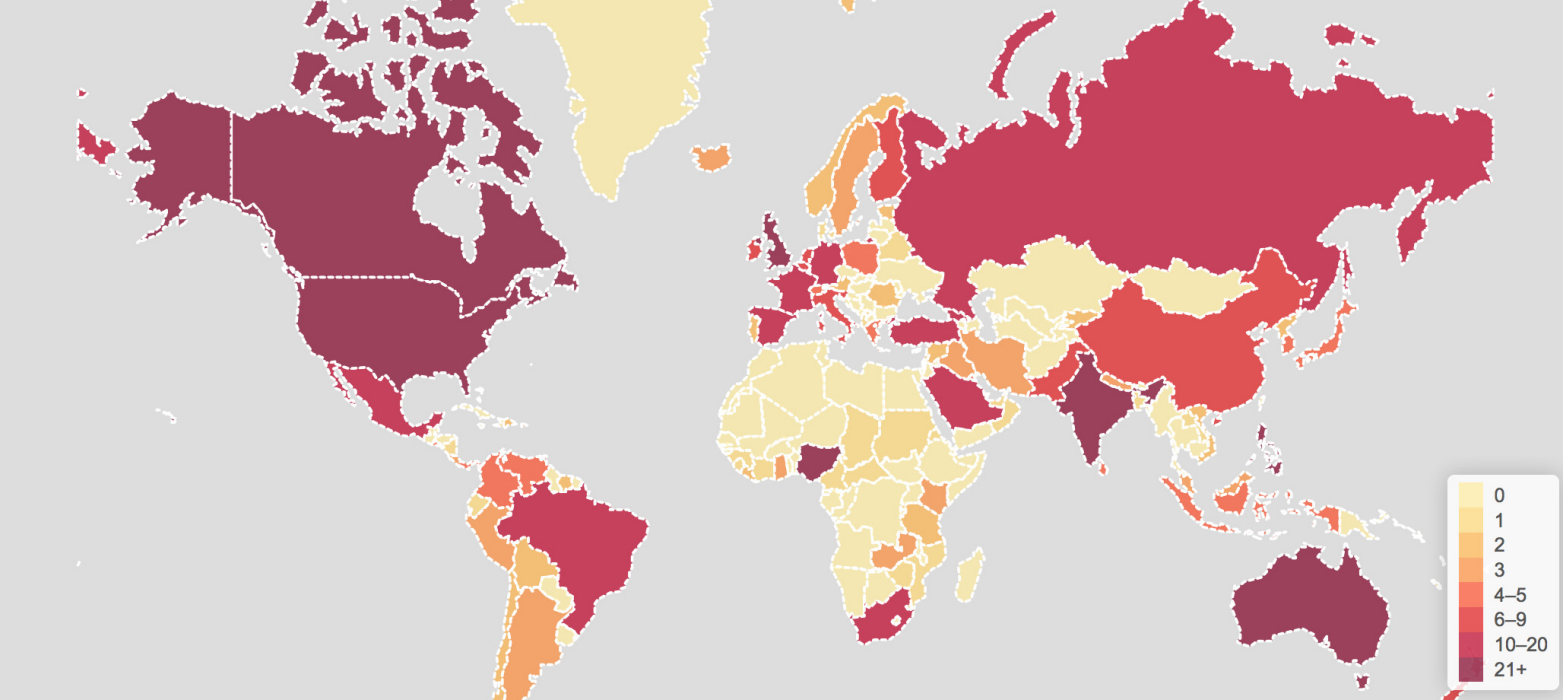
Geolocation of users contributing to the #KidneyStones online discussion on Twitter. The colour tone reflects the number of users per country: the colour shifts from light tones (countries with few or no users) to dark tones (countries with many users).

**Fig 4:**
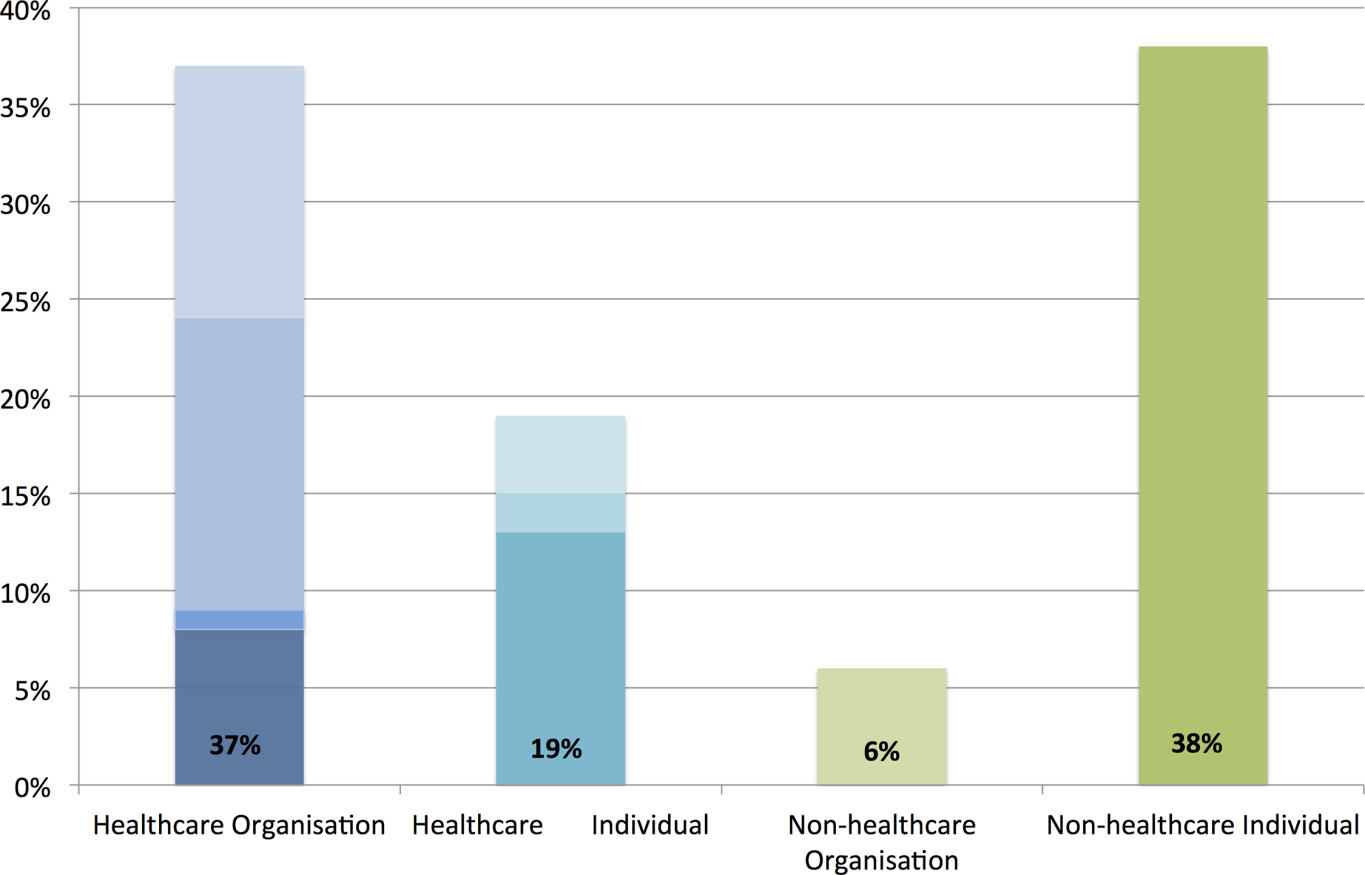
Top 100 influencers in the #KidneyStones online discussion on Twitter according to tweet volume stratified by healthcare category. Healthcare organisation consists of 8% provider, 1% government, 15% advocate/support and 13% other healthcare organisations (from darker blue to lighter blue). Healthcare individual consists of 13% doctors, 2% healthcare practitioners and 4% other healthcare individuals (from darker turquoise to lighter turquoise).

Fig 2 demonstrates that the monthly number of users contributing to the #KidneyStones online discussion on Twitter from October 2014 grew steadily, with a median of 262 (range 189–454) new users per month. The median number of active users contributing to the #KidneyStones online discussion was 304.5 per month (range 227–545). Users came from 106 countries and from all continents around the globe (Fig 3). The S1 Table lists the location of users according to country and continent. North American users were most active (63%), ahead of European users (16%) and Asian users (10%). The top 100 influencers in the #KidneyStones online discussion on Twitter, according to the number of tweets posted in the study period, and stratified by healthcare category, are shown in Fig 4. Individuals not involved in healthcare (38%) and healthcare organisations (37%; organisations: provider, advocate/support, government, other) were the main influencers in the discussion. There were no spam accounts among the top 100 influencers.

**Fig 5:**
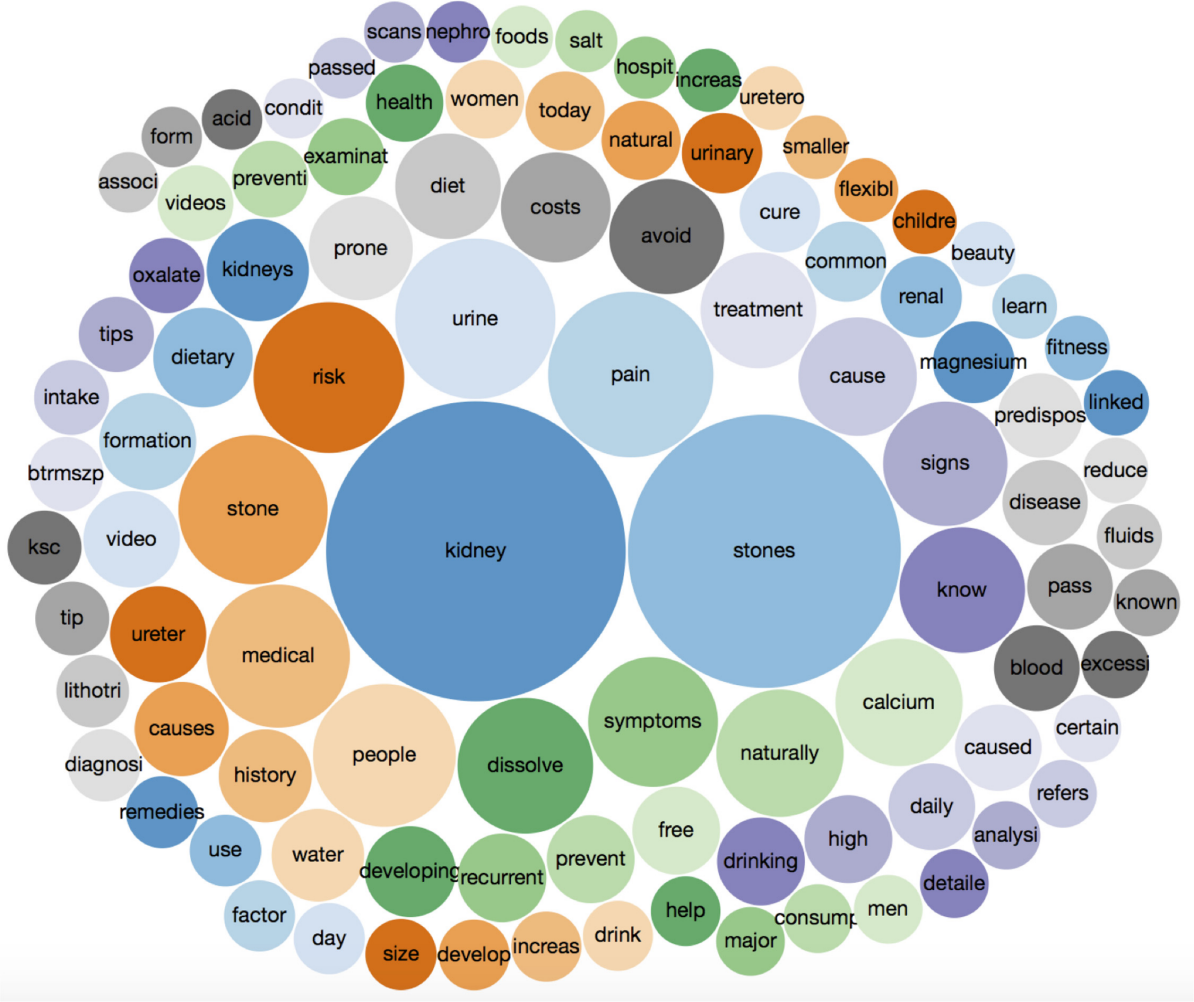
Bubble chart visualising the 100 most frequently used words in tweets in the #KidneyStones online discussion on Twitter. The larger the bubble, the more a word is used. Colours only aid differentiation and have no other significance.

A content analysis of the most frequently used words in the #KidneyStones online discussion on Twitter is shown in Fig 5. Besides ‘kidney’ (used 4,045 times) and ‘stones’ (3,335), ‘pain’ (1,233), ‘urine’ (1,158), and ‘risk’ (1,023) were the top words. The S2 Table presents the complete content analysis with the 100 most frequently used words in #KidneyStones tweets. The largest thematic proportion of tweets focused on disease awareness and prevention (35 of 100 words). The sentiment analysis revealed that 56% of the tweets had a positive sentiment and 44% had a negative sentiment. The median (range) number of shares for the top 10 links was 85 (62–587) and 45.5 (17–94) for the top 10 photos. The top 10 retweets had a median of 44 (22–95) retweets. The #KidneyStones hashtag was often related to the #health, #renalcolic, #renalcalculus and #kidney hashtags.

## Discussion

We performed a longitudinal assessment of activity, users and content of the online discussion on #KidneyStones, using the Symplur Signals health analytics database. Over the one year period, Twitter activity was reflected by 10,806 tweets, which were frequently enhanced with links and mentions, and were posted predominantly in English. Together, 3,426 users from 106 countries contributed to the online discussion, with non-professional individuals and healthcare organisations being the main influencers. Content was dominated by the words ‘kidney’, ‘stones’, ‘pain’, ‘urine’ and ‘risk’, and tweets had more positive than negative sentiments. The top links, photos and retweets were shared up to 100 times.

The Twitter activity of 10,806 tweets found for #KidneyStones in the study period is much higher than 880 tweets found in 2012 for urinary tract infection, another urological disease [14]. The majority of Twitter activity data in the literature are published on urological oncology diseases, such as #testicularcancer (10,376 tweets in 2014) or #prostatecancer (79,242 tweets in 2014) [15]. Comparisons of tweet activity between #KidneyStones as a benign disease and malignant diseases are compromised since the potentially life-threatening character of oncological topics has been shown to provoke an over-representation on Twitter [14].

The tweet metric analysis showed that contributors interacted using retweets (24%) and mentions (30%) in their tweets. Relevant tweets inducing retweets are of high interest to the Twitter community, which is underlined by the recently introduced concept of the Twitter impact factor [16]. Moreover, almost half of the tweets were enhanced by a link. This is encouraging since the combination ‘statement + proof’ is a basic principle in science which can be translated to Twitter as the combination ‘statement + link’. A tweet analysis on the topic of dementia showed that the top users applied links more frequently than average users [17]. Similarly, successful tweets from public state health departments contained links in the majority of tweets [18]. Thus, a high number of links used in tweets for #KidneyStones appears to enhance the reach of tweets and enables followers to check the trustworthiness of the short information presented in one tweet. Additionally, links enable more information to be conveyed in the tweet than the 140 characters would otherwise allow.

The Twitter discussion on #KidneyStones is global, involving users from all continents. Usage rates of social media were 74% among American urologists in 2013 [19] and 70% among Australian urologists in 2014 [1]. As social media adoption rates continue to grow, so did the number of cumulative contributors to the #KidneyStones discussion. This is in line with our previous study on urologic oncology, with steadily increasing tweet activity over time [15]. Twitter allows for rapid, informal, and thus low-threshold, participation in an online discussion. These characteristics make it appealing for laypeople to join in the conversation. Notably, individuals such as patients, relatives and interested people who are not primarily involved in healthcare accounted for a large number of the top influencers in the #KidneyStones discussion. Similarly, individual and organisational healthcare professionals contributed as top influencers, which demonstrates the potential of Twitter for disseminating valuable knowledge from professionals on preventive, diagnostic and therapeutic options to the populace.

Particularly noteworthy is the finding of the content analysis, revealing that words symbolising disease awareness and prevention are most frequently used in tweets on #KidneyStones. Primary prevention of kidney stones has been shown to be cost-effective for a national healthcare system [20]. This is crucial, particularly considering the rapidly increasing costs of urolithiasis treatment, predicted to be more than US$1 billion annually in the United States by 2030 [9]. Importantly, urolithiasis is associated with a 30–50% risk of recurrence within seven years of initial treatment [21, 22]. Secondary nonmedical prevention with fluid intake, specific dietary therapy, adoption of a ‘healthy’ lifestyle [23–25] as well as preventive pharmacological treatment [26–28] were reported to effectively reduce recurrence rates. Unfortunately, due to patient and provider scepticism about the evidence of secondary prevention effectiveness, it is infrequently utilised in daily routines [29, 30]. An additional hurdle to the implementation of preventive measures is low patient compliance; roughly half of stone formers were reported to adhere to a prescribed preventive therapy in a contemporary series [30]. Considering the under-utilised potential of primary and secondary prevention of urolithiasis, the Twitter platform might make a beneficial contribution in these areas. The #KidneyStones Twitter discussion can deliver currently valid guidelines and recommendations on urolithiasis prevention to laypeople and thus lead to both a decrease of recurrence rates and increased cost-savings. In this context, using links and photos to enhance a tweet’s content and its reach can therefore be a successful strategy. The most shared link in our analysis was distributed 587 times and the most shared photos and retweets were spread up to nearly 100 times.

Although the #KidneyStones hashtag is proposed as standardised communication descriptor [5], we acknowledge that this single hashtag cannot capture all the information that is exchanged on urolithiasis on the Twitter platform. Particularly during congresses with high tweet activity using multiple hashtags, discussions on stone disease might take place beside the #KidneyStones channel.

We also used the Symplur Signals analytics platform for a systematic assessment of healthcare social media data. The automated data extraction and analysis algorithms allow for the analysis of a vast amount of data, but cannot detect linguistic nuances, such as ambiguity or irony when analysing content. Lastly, Twitter is a rapidly growing and changing social media platform, implying that the results of our contemporary analysis might be out-dated in the near future.

Notwithstanding the aforementioned limitations, several practice-oriented conclusions can be drawn from the findings of the current study. The Twitter discussion on #KidneyStones is maintained by users from all over the world and evokes a remarkable number of tweets, underscoring the global reach of this microblogging platform. Healthcare organisations, as one of the top influencers in the discussion, have a unique opportunity to raise the awareness of patients and providers for nonmedical and pharmacological prevention, eventually reducing recurrence rates and care-related expenditure. Patients and other laypeople substantially contributing to the discussion have the option of being discreetly and noncommittally counselled by experts, optimising shared decision-making. Finally, given responsible Twitter usage, the dissemination of novel diagnostic and therapeutic developments in the area of urolithiasis between stakeholders and patients might be considerably accelerated.

## Conclusion

The Twitter discussion on #KidneyStones engaged multiple stakeholders in the healthcare sector on a global scale and involves both professionals and laypeople. Considering the rapidly increasing prevalence and treatment-related costs of urolithiasis, Twitter might promote shared decision-making and contribute to the optimisation of patient care.

## Supporting Information

S1 Table Geolocation of users of the #KidneyStones online discussion on Twitter. (XLSX)

S2 Table Top 100 words used in the #KidneyStones online discussion on Twitter. (XLSX)
